# 4-Nitro-*N*-phthalyl-l-tryptophan

**DOI:** 10.1107/S1600536811029138

**Published:** 2011-07-23

**Authors:** Anaelle Tilborg, Irving Boittiaux, Bernadette Norberg, Didier Lambert, Johan Wouters

**Affiliations:** a Department of Chemistry, University of Namur, 61, Rue de Bruxelles, B-5000 Namur, Belgium; b Louvain Drug Research Institute (LDRI), UCL, 50, Avenue Mounier, B-1200 Woluwe-Saint-Lambert, Belgium

## Abstract

The crystal structure of the title compound [systematic name: (2*R*)-3-(1*H*-indol-3-yl)-2-(4-nitro-1,3-dioxoisoindolin-2-yl)propanoic acid], C_19_H_13_N_3_O_6_, an analogue of epigenetic modulator RG108, is constrained by strong hydrogen bonds between the indole N—H group and a carbonyl O atom of the phthalimide ring of a symmetry-related mol­ecule, and between the protonated O atom of the carboxyl group and a carbonyl O atom of the phthalimide ring. π–π stacking inter­actions with centroid–centroid distances of 3.638 (1) and 3.610 (1) Å are also observed between indole and phthalimide rings.

## Related literature

For crystallographic information and details of the RG108 analogue, see: Braun *et al.* (2010 ▶) and for details of the biological evaluation, see: Brueckner *et al.* (2005 ▶).
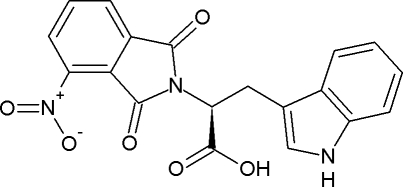


## Experimental

### 

#### Crystal data

C_19_H_13_N_3_O_6_
*M* *_r_* = 379.33
Monoclinic, 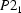

*a* = 7.0569 (3) Å
*b* = 15.5302 (8) Å
*c* = 7.6947 (4) Å
β = 95.415 (4)°
*V* = 839.54 (7) Å^3^
*Z* = 2
Cu *K*α radiation
μ = 0.97 mm^−1^
*T* = 293 K
0.22 × 0.10 × 0.03 mm

#### Data collection

Oxford Diffraction Xcalibur Ruby Gemini ultra diffractometer
Absorption correction: multi-scan (*CrysAlis PRO*; Oxford Diffraction, 2009 ▶) *T*_min_ = 0.815, *T*_max_ = 0.972
9007 measured reflections
2966 independent reflections
2735 reflections with *I* > 2σ(*I*)
*R*_int_ = 0.029

#### Refinement

*R*[*F* ^2^ > 2σ(*F* ^2^)] = 0.031
*wR*(*F* ^2^) = 0.091
*S* = 1.06
2966 reflections
262 parameters
1 restraint
H atoms treated by a mixture of independent and constrained refinement
Δρ_max_ = 0.15 e Å^−3^
Δρ_min_ = −0.13 e Å^−3^
Absolute structure: Flack (1983 ▶), 1371 Friedel pairs
Flack parameter: −0.1 (2)

### 

Data collection: *CrysAlis PRO* (Oxford Diffraction, 2009 ▶); cell refinement: *CrysAlis PRO*; data reduction: *CrysAlis PRO*; program(s) used to solve structure: *SIR92* (Altomare *et al.*, 1994 ▶); program(s) used to refine structure: *SHELXL97* (Sheldrick, 2008 ▶); molecular graphics: *ORTEPIII* (Burnett & Johnson, 1996 ▶) and *PLATON* (Spek, 2009 ▶); software used to prepare material for publication: *SHELXL97*.

## Supplementary Material

Crystal structure: contains datablock(s) I, global. DOI: 10.1107/S1600536811029138/vm2109sup1.cif

Structure factors: contains datablock(s) I. DOI: 10.1107/S1600536811029138/vm2109Isup2.hkl

Supplementary material file. DOI: 10.1107/S1600536811029138/vm2109Isup3.cml

Additional supplementary materials:  crystallographic information; 3D view; checkCIF report

## Figures and Tables

**Table 1:** Hydrogen-bond geometry (Å, °)

*D*—H⋯*A*	*D*—H	H⋯*A*	*D*⋯*A*	*D*—H⋯*A*
N2—H2⋯O1^i^	0.86 (4)	2.28 (4)	3.002 (3)	142 (4)
O4—H1⋯O2^ii^	1.00 (4)	1.78 (4)	2.716 (2)	154 (3)

Symmetry codes: (i) 
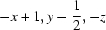
; (ii) 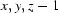.

**Fig. 1.**
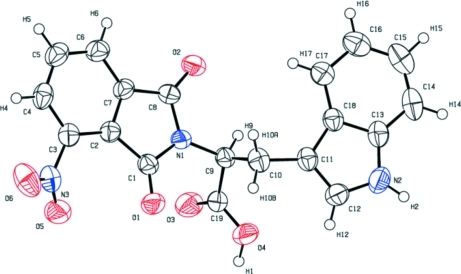
ORTEP view and atom numbering of the title compound. Displacement ellipsoids for non-H atoms are drawn at the 50% probability level.

## supplementary crystallographic information

### Comment

4-Nitro-*N*-phthalyl-L-tryptophan is an analog of RG108, a DNA methyltransferase (DNMT) inhibitor discovered by virtual screening (Brueckner *et al.* (2005)). Introduction of a nitro functional group on the phtalimide moiety is excepted to improve inhibition ability (unpublished results).

Isomer *S* (C9) of 4-nitro-*N*-phthalyl-tryptophan is obtained from L-tryptophan and nitrophthalic anhydride in DMF.

Two main types of H-bonding interactions are observed in the structure: one between N—H of the indole ring (N2) and O1 of the phtalimide ring, and the other between the protonated oxygen (O4) from the carboxylic moiety and O2 from the phtalimide ring (see Table 1).

In addition to H-bonds, crystal packing organization is further stabilized by π–π-stacking interactions involving symmetry-related molecules, in particularly between the 6-membered coupled rings of nitrophthalimide and indole moiety (see Table 2). No interactions of this type are present in the packing of the dicyclohexylamine salt of RG108 (Braun *et al.* (2010), because of the presence of the ammonium counter-cation and water molecules included in the crystalline network.

In contrast to the structure of the dicyclohexylamine salt of RG108 (Braun *et al.* (2010)), where the compound conformation is constrained by strong (charge-assisted) H-bonds with the dicyclohexylammonium ion and extra water molecules, the angle between the two fused rings is 14.23 (4)° in the present structure compared to 58.35 (4)° in the case of the RG108 salt. The torsion angle of the chain between the two aromatic moieties (N1—C9—C10—C11) is also distinct: -155.66 (17)° and -61.93 (17) ° for the title and RG108 salt structures, respectively.

### Experimental

Synthesis of the compound was accomplished by micro-wave heating of L-tryptophan (1 mmol, 204 mg) and 4-nitro phthalic anhydride (1 mmol, 193 mg) in 5 ml of DMF. The mixture was then poured in cold aqueous buffer solution (pH = 2) and extracted with ethyl acetate. After drying with Na_2_SO_4_, the organic phase is evaporated and the residue is purified by flash chromatography (dichloromethane and methanol: 9/1; yield = 60%, 230 mg).

### Refinement

H1 and H2, bound to O4 and N2 respectively and involved in hydrogen bonds, were located from ΔF Fourier difference maps and their position refined freely. All other remaining H-atoms were placed at idealized positions and allowed to ride on their parent atoms, with C—H distances of 0.93 – 0.98 Å and with *U*_iso_(H) = 1.2*U*_eq_(C).

### Crystal data

**Table:**

C_19_H_13_N_3_O_6_	*F*(000) = 392
*M**_r_* = 379.33	*D*_x_ = 1.501 Mg m^−^^3^
Monoclinic, *P*2_1_	Cu *K*α radiation, λ = 1.54184 Å
Hall symbol: P 2yb	Cell parameters from 4268 reflections
*a* = 7.0569 (3) Å	θ = 2.8–67.9°
*b* = 15.5302 (8) Å	µ = 0.97 mm^−^^1^
*c* = 7.6947 (4) Å	*T* = 293 K
β = 95.415 (4)°	Prism, yellow
*V* = 839.54 (7) Å^3^	0.22 × 0.10 × 0.03 mm
*Z* = 2	

### Data collection

**Table:**

Oxford Diffraction Xcalibur Ruby Gemini ultra diffractometer	2966 independent reflections
Radiation source: fine-focus sealed tube	2735 reflections with *I* > 2σ(*I*)
graphite	*R*_int_ = 0.029
Detector resolution: 10.3712 pixels mm^-1^	θ_max_ = 68.0°, θ_min_ = 5.7°
ω scans	*h* = −8→8
Absorption correction: multi-scan (*CrysAlis PRO*; Oxford Diffraction, 2009)	*k* = −18→17
*T*_min_ = 0.815, *T*_max_ = 0.972	*l* = −9→8
9007 measured reflections	

### Refinement

**Table:**

Refinement on *F*^2^	Hydrogen site location: inferred from neighbouring sites
Least-squares matrix: full	H atoms treated by a mixture of independent and constrained refinement
*R*[*F*^2^ > 2σ(*F*^2^)] = 0.031	*w* = 1/[σ^2^(*F*_o_^2^) + (0.0492*P*)^2^ + 0.1477*P*] where *P* = (*F*_o_^2^ + 2*F*_c_^2^)/3
*wR*(*F*^2^) = 0.091	(Δ/σ)_max_ < 0.001
*S* = 1.06	Δρ_max_ = 0.15 e Å^−^^3^
2966 reflections	Δρ_min_ = −0.13 e Å^−^^3^
262 parameters	Extinction correction: *SHELXL97* (Sheldrick, 2008), Fc^*^=kFc[1+0.001xFc^2^λ^3^/sin(2θ)]^-1/4^
1 restraint	Extinction coefficient: 0.0080 (8)
0 constraints	Absolute structure: Flack (1983), 1371 Friedel pairs
Primary atom site location: structure-invariant direct methods	Flack parameter: −0.1 (2)
Secondary atom site location: difference Fourier map	

### Special details

**Table:**

Geometry. All e.s.d.'s (except the e.s.d. in the dihedral angle between two l.s. planes) are estimated using the full covariance matrix. The cell e.s.d.'s are taken into account individually in the estimation of e.s.d.'s in distances, angles and torsion angles; correlations between e.s.d.'s in cell parameters are only used when they are defined by crystal symmetry. An approximate (isotropic) treatment of cell e.s.d.'s is used for estimating e.s.d.'s involving l.s. planes.
Refinement. Refinement of *F*^2^ against ALL reflections. The weighted *R*-factor *wR* and goodness of fit *S* are based on *F*^2^, conventional *R*-factors *R* are based on *F*, with *F* set to zero for negative *F*^2^. The threshold expression of *F*^2^ > σ(*F*^2^) is used only for calculating *R*-factors(gt) *etc*. and is not relevant to the choice of reflections for refinement. *R*-factors based on *F*^2^ are statistically about twice as large as those based on *F*, and *R*- factors based on ALL data will be even larger.

### Fractional atomic coordinates and isotropic or equivalent isotropic displacement parameters (Å^2^)

**Table:**

	*x*	*y*	*z*	*U*_iso_*/*U*_eq_	
C1	0.7937 (3)	0.58969 (13)	0.3293 (3)	0.0401 (4)	
C2	0.8237 (3)	0.64858 (13)	0.4829 (3)	0.0385 (4)	
C3	0.7949 (3)	0.73572 (14)	0.5072 (3)	0.0427 (5)	
C4	0.8157 (3)	0.77052 (15)	0.6745 (3)	0.0481 (5)	
H4	0.7881	0.8283	0.6913	0.058*	
C5	0.8772 (3)	0.71950 (17)	0.8159 (3)	0.0533 (6)	
H5	0.8943	0.7437	0.9269	0.064*	
C6	0.9135 (3)	0.63265 (16)	0.7939 (3)	0.0497 (5)	
H6	0.9581	0.5984	0.8881	0.060*	
C7	0.8815 (3)	0.59866 (14)	0.6286 (3)	0.0410 (4)	
C8	0.8976 (3)	0.50848 (14)	0.5709 (2)	0.0402 (4)	
C9	0.8329 (3)	0.42945 (12)	0.2879 (3)	0.0391 (4)	
H9	0.9070	0.3854	0.3557	0.047*	
C10	0.6282 (3)	0.39570 (14)	0.2619 (3)	0.0479 (5)	
H10A	0.5655	0.4077	0.3661	0.057*	
H10B	0.5601	0.4265	0.1656	0.057*	
C11	0.6167 (3)	0.30101 (14)	0.2249 (3)	0.0437 (5)	
C12	0.5649 (3)	0.26268 (17)	0.0691 (3)	0.0532 (6)	
H12	0.5355	0.2917	−0.0357	0.064*	
C13	0.6095 (3)	0.15405 (15)	0.2600 (3)	0.0488 (5)	
C14	0.6192 (3)	0.07499 (16)	0.3441 (4)	0.0607 (7)	
H14	0.5920	0.0241	0.2830	0.073*	
C15	0.6705 (3)	0.07453 (17)	0.5213 (4)	0.0650 (7)	
H15	0.6779	0.0224	0.5810	0.078*	
C16	0.7115 (3)	0.15063 (19)	0.6124 (4)	0.0590 (6)	
H16	0.7468	0.1482	0.7318	0.071*	
C17	0.7012 (3)	0.22977 (16)	0.5303 (3)	0.0479 (5)	
H17	0.7294	0.2801	0.5928	0.058*	
C18	0.6472 (3)	0.23231 (14)	0.3505 (3)	0.0413 (4)	
C19	0.9328 (3)	0.44380 (13)	0.1227 (3)	0.0433 (5)	
N1	0.8488 (2)	0.50773 (11)	0.3922 (2)	0.0397 (4)	
N2	0.5622 (3)	0.17470 (15)	0.0885 (3)	0.0591 (6)	
N3	0.7496 (3)	0.79406 (12)	0.3599 (3)	0.0518 (5)	
O1	0.7311 (2)	0.60263 (10)	0.18047 (19)	0.0526 (4)	
O2	0.9409 (2)	0.44385 (10)	0.65541 (19)	0.0518 (4)	
O3	1.0857 (3)	0.47711 (14)	0.1246 (2)	0.0659 (5)	
O4	0.8377 (3)	0.41420 (14)	−0.0186 (2)	0.0709 (6)	
O5	0.8221 (3)	0.77922 (12)	0.2254 (2)	0.0683 (5)	
O6	0.6475 (3)	0.85593 (12)	0.3807 (3)	0.0768 (6)	
H1	0.913 (5)	0.425 (2)	−0.120 (5)	0.093 (11)*	
H2	0.525 (6)	0.138 (3)	0.008 (5)	0.095 (12)*	

### Atomic displacement parameters (Å^2^)

**Table:**

	*U*^11^	*U*^22^	*U*^33^	*U*^12^	*U*^13^	*U*^23^
C1	0.0449 (10)	0.0354 (11)	0.0400 (11)	0.0023 (8)	0.0046 (8)	0.0025 (8)
C2	0.0382 (9)	0.0391 (11)	0.0391 (10)	−0.0004 (8)	0.0080 (8)	−0.0029 (8)
C3	0.0408 (10)	0.0409 (11)	0.0475 (11)	−0.0011 (8)	0.0092 (8)	−0.0035 (9)
C4	0.0440 (11)	0.0449 (12)	0.0576 (13)	−0.0067 (9)	0.0161 (9)	−0.0150 (10)
C5	0.0539 (12)	0.0625 (14)	0.0451 (12)	−0.0119 (11)	0.0133 (10)	−0.0138 (11)
C6	0.0527 (12)	0.0579 (15)	0.0397 (11)	−0.0068 (10)	0.0100 (9)	−0.0027 (10)
C7	0.0434 (10)	0.0442 (11)	0.0363 (10)	−0.0038 (8)	0.0083 (8)	−0.0055 (8)
C8	0.0414 (10)	0.0436 (11)	0.0362 (10)	0.0003 (8)	0.0078 (8)	0.0037 (9)
C9	0.0482 (10)	0.0314 (10)	0.0381 (10)	0.0020 (8)	0.0068 (8)	0.0011 (8)
C10	0.0477 (12)	0.0417 (12)	0.0549 (13)	0.0001 (9)	0.0085 (9)	0.0007 (10)
C11	0.0409 (10)	0.0400 (11)	0.0511 (12)	−0.0037 (8)	0.0087 (9)	−0.0006 (9)
C12	0.0575 (13)	0.0598 (15)	0.0422 (11)	−0.0117 (11)	0.0034 (10)	−0.0017 (10)
C13	0.0442 (11)	0.0413 (11)	0.0618 (14)	−0.0044 (9)	0.0104 (10)	−0.0042 (10)
C14	0.0490 (12)	0.0396 (12)	0.095 (2)	−0.0061 (9)	0.0157 (13)	−0.0023 (12)
C15	0.0497 (12)	0.0517 (15)	0.095 (2)	0.0012 (11)	0.0143 (13)	0.0218 (14)
C16	0.0486 (12)	0.0678 (16)	0.0610 (15)	0.0017 (11)	0.0068 (10)	0.0146 (12)
C17	0.0430 (10)	0.0505 (12)	0.0506 (12)	0.0002 (9)	0.0060 (9)	0.0034 (10)
C18	0.0371 (9)	0.0404 (11)	0.0474 (11)	−0.0035 (8)	0.0088 (8)	−0.0003 (9)
C19	0.0553 (12)	0.0359 (10)	0.0388 (10)	−0.0026 (9)	0.0053 (9)	−0.0012 (8)
N1	0.0527 (10)	0.0349 (9)	0.0315 (8)	0.0015 (7)	0.0053 (7)	−0.0001 (7)
N2	0.0653 (13)	0.0558 (14)	0.0568 (13)	−0.0161 (10)	0.0081 (10)	−0.0152 (10)
N3	0.0536 (10)	0.0382 (10)	0.0639 (13)	−0.0008 (8)	0.0069 (9)	−0.0001 (8)
O1	0.0717 (10)	0.0440 (8)	0.0401 (8)	0.0082 (7)	−0.0046 (7)	−0.0003 (7)
O2	0.0694 (10)	0.0456 (9)	0.0406 (8)	0.0043 (7)	0.0057 (7)	0.0065 (7)
O3	0.0628 (11)	0.0857 (13)	0.0511 (9)	−0.0194 (9)	0.0148 (8)	−0.0044 (8)
O4	0.0945 (13)	0.0828 (13)	0.0363 (8)	−0.0362 (11)	0.0113 (9)	−0.0052 (8)
O5	0.1017 (14)	0.0533 (10)	0.0511 (10)	0.0083 (9)	0.0135 (9)	0.0002 (8)
O6	0.0812 (12)	0.0447 (10)	0.1084 (16)	0.0170 (9)	0.0289 (11)	0.0126 (10)

### Geometric parameters (Å, °)

**Table:**

C1—O1	1.205 (3)	C10—H10B	0.9700
C1—N1	1.403 (3)	C11—C12	1.358 (3)
C1—C2	1.493 (3)	C11—C18	1.442 (3)
C2—C3	1.384 (3)	C12—N2	1.375 (3)
C2—C7	1.392 (3)	C12—H12	0.9300
C3—C4	1.391 (3)	C13—N2	1.369 (3)
C3—N3	1.463 (3)	C13—C14	1.386 (4)
C4—C5	1.382 (4)	C13—C18	1.413 (3)
C4—H4	0.9300	C14—C15	1.377 (4)
C5—C6	1.386 (4)	C14—H14	0.9300
C5—H5	0.9300	C15—C16	1.391 (4)
C6—C7	1.376 (3)	C15—H15	0.9300
C6—H6	0.9300	C16—C17	1.381 (4)
C7—C8	1.477 (3)	C16—H16	0.9300
C8—O2	1.219 (2)	C17—C18	1.401 (3)
C8—N1	1.386 (3)	C17—H17	0.9300
C9—N1	1.455 (2)	C19—O3	1.195 (3)
C9—C19	1.527 (3)	C19—O4	1.306 (3)
C9—C10	1.532 (3)	N2—H2	0.86 (4)
C9—H9	0.9800	N3—O5	1.219 (3)
C10—C11	1.499 (3)	N3—O6	1.221 (3)
C10—H10A	0.9700	O4—H1	1.00 (4)
			
O1—C1—N1	122.92 (18)	C12—C11—C10	127.1 (2)
O1—C1—C2	131.44 (18)	C18—C11—C10	126.7 (2)
N1—C1—C2	105.61 (16)	C11—C12—N2	110.2 (2)
C3—C2—C7	118.11 (19)	C11—C12—H12	124.9
C3—C2—C1	133.98 (19)	N2—C12—H12	124.9
C7—C2—C1	107.76 (18)	N2—C13—C14	130.9 (2)
C2—C3—C4	120.1 (2)	N2—C13—C18	106.9 (2)
C2—C3—N3	121.67 (19)	C14—C13—C18	122.2 (2)
C4—C3—N3	118.2 (2)	C15—C14—C13	117.6 (2)
C5—C4—C3	120.2 (2)	C15—C14—H14	121.2
C5—C4—H4	119.9	C13—C14—H14	121.2
C3—C4—H4	119.9	C14—C15—C16	121.2 (2)
C4—C5—C6	120.6 (2)	C14—C15—H15	119.4
C4—C5—H5	119.7	C16—C15—H15	119.4
C6—C5—H5	119.7	C17—C16—C15	121.8 (2)
C7—C6—C5	118.1 (2)	C17—C16—H16	119.1
C7—C6—H6	121.0	C15—C16—H16	119.1
C5—C6—H6	121.0	C16—C17—C18	118.3 (2)
C6—C7—C2	122.7 (2)	C16—C17—H17	120.8
C6—C7—C8	129.2 (2)	C18—C17—H17	120.8
C2—C7—C8	108.11 (17)	C17—C18—C13	118.9 (2)
O2—C8—N1	123.29 (19)	C17—C18—C11	133.8 (2)
O2—C8—C7	129.99 (18)	C13—C18—C11	107.29 (19)
N1—C8—C7	106.71 (17)	O3—C19—O4	123.7 (2)
N1—C9—C19	108.63 (16)	O3—C19—C9	122.70 (19)
N1—C9—C10	112.37 (17)	O4—C19—C9	113.59 (18)
C19—C9—C10	116.40 (17)	C8—N1—C1	111.65 (16)
N1—C9—H9	106.3	C8—N1—C9	123.58 (16)
C19—C9—H9	106.3	C1—N1—C9	124.30 (16)
C10—C9—H9	106.3	C13—N2—C12	109.5 (2)
C11—C10—C9	113.22 (18)	C13—N2—H2	125 (3)
C11—C10—H10A	108.9	C12—N2—H2	125 (3)
C9—C10—H10A	108.9	O5—N3—O6	124.1 (2)
C11—C10—H10B	108.9	O5—N3—C3	117.55 (18)
C9—C10—H10B	108.9	O6—N3—C3	118.3 (2)
H10A—C10—H10B	107.7	C19—O4—H1	109 (2)
C12—C11—C18	106.1 (2)		
			
O1—C1—C2—C3	−1.5 (4)	C16—C17—C18—C13	−1.3 (3)
N1—C1—C2—C3	−179.2 (2)	C16—C17—C18—C11	178.1 (2)
O1—C1—C2—C7	173.8 (2)	N2—C13—C18—C17	−179.45 (18)
N1—C1—C2—C7	−3.9 (2)	C14—C13—C18—C17	1.8 (3)
C7—C2—C3—C4	−2.8 (3)	N2—C13—C18—C11	1.0 (2)
C1—C2—C3—C4	172.1 (2)	C14—C13—C18—C11	−177.7 (2)
C7—C2—C3—N3	174.80 (18)	C12—C11—C18—C17	−179.8 (2)
C1—C2—C3—N3	−10.3 (3)	C10—C11—C18—C17	−3.3 (4)
C2—C3—C4—C5	4.2 (3)	C12—C11—C18—C13	−0.4 (2)
N3—C3—C4—C5	−173.46 (19)	C10—C11—C18—C13	176.2 (2)
C3—C4—C5—C6	−1.9 (3)	N1—C9—C19—O3	45.1 (3)
C4—C5—C6—C7	−1.7 (3)	C10—C9—C19—O3	173.1 (2)
C5—C6—C7—C2	3.2 (3)	N1—C9—C19—O4	−136.7 (2)
C5—C6—C7—C8	−176.1 (2)	C10—C9—C19—O4	−8.7 (3)
C3—C2—C7—C6	−0.9 (3)	O2—C8—N1—C1	176.27 (19)
C1—C2—C7—C6	−177.10 (18)	C7—C8—N1—C1	−2.7 (2)
C3—C2—C7—C8	178.52 (17)	O2—C8—N1—C9	3.8 (3)
C1—C2—C7—C8	2.4 (2)	C7—C8—N1—C9	−175.13 (18)
C6—C7—C8—O2	0.6 (4)	O1—C1—N1—C8	−173.90 (19)
C2—C7—C8—O2	−178.8 (2)	C2—C1—N1—C8	4.1 (2)
C6—C7—C8—N1	179.5 (2)	O1—C1—N1—C9	−1.5 (3)
C2—C7—C8—N1	0.1 (2)	C2—C1—N1—C9	176.45 (17)
N1—C9—C10—C11	−155.69 (18)	C19—C9—N1—C8	−134.89 (18)
C19—C9—C10—C11	78.1 (2)	C10—C9—N1—C8	94.9 (2)
C9—C10—C11—C12	−105.8 (3)	C19—C9—N1—C1	53.6 (2)
C9—C10—C11—C18	78.3 (3)	C10—C9—N1—C1	−76.7 (2)
C18—C11—C12—N2	−0.4 (3)	C14—C13—N2—C12	177.3 (2)
C10—C11—C12—N2	−176.9 (2)	C18—C13—N2—C12	−1.3 (3)
N2—C13—C14—C15	−179.5 (2)	C11—C12—N2—C13	1.1 (3)
C18—C13—C14—C15	−1.1 (3)	C2—C3—N3—O5	−34.6 (3)
C13—C14—C15—C16	−0.1 (4)	C4—C3—N3—O5	143.1 (2)
C14—C15—C16—C17	0.5 (4)	C2—C3—N3—O6	147.9 (2)
C15—C16—C17—C18	0.2 (3)	C4—C3—N3—O6	−34.5 (3)

### Hydrogen-bond geometry (Å, °)

**Table:**

*D*—H···*A*	*D*—H	H···*A*	*D*···*A*	*D*—H···*A*
N2—H2···O1^i^	0.86 (4)	2.28 (4)	3.002 (3)	142 (4)
O4—H1···O2^ii^	1.00 (4)	1.78 (4)	2.716 (2)	154 (3)

Symmetry codes: (i) −*x*+1, *y*−1/2, −*z*; (ii) *x*, *y*, *z*−1.

### Table 2 π–π stacking interactions between six-membered rings from indole (C13—C18; ring centroid Cg(1)) and nitrophthalimide (C2—C7; ring centroid Cg(2)).

Cg-Cg : distance (Å) between ring centroids; α : dihedral angle(°) between ring planes 1 and 2 ; β : angle (°) between Cg(1)-->Cg(2) vector and normal to ring plane 1 ; γ : angle (°) between Cg(1)-->Cg(2) vector and normal to ring plane 2 ; Cg1_Perp  : perpendicular distance (Å) of Cg(1) on ring plane 2 ; Cg2_Perp  : perpendicular distance (Å) of Cg(2) on ring plane 1.

**Table:**

Cg(I)-Cg(J)	sym (J)	Cg-Cg	α	β	γ	Cg1_Perp	Cg2_Perp
Cg(1)-Cg(2)	(i)	3.638 (1)	7.24	18.69	20.72	-3.403 (1)	3.446 (1)
Cg(1)-Cg(2)	(ii)	3.610 (1)	7.24	19.41	12.26	3.528 (1)	3.405 (1)

Symmetry codes : (i) 1 - x, 1/2 + y, 1 - z, (ii) 2 - x, 1/2 + y, 1 - z
